# Histopathological and clinical characteristics of duodenal gastrointestinal stromal tumors as predictors of malignancy

**DOI:** 10.1186/1477-7819-11-202

**Published:** 2013-08-16

**Authors:** Tsunenori Saito, Masaki Ueno, Yasunori Ota, Yoshiharu Nakamura, Masaji Hashimoto, Harushi Udagawa, Kyoichi Mizuno, Kenichi Ohashi, Goro Watanabe

**Affiliations:** 1 Department of Internal Medicine, Nippon Medical School, 1-1-5 Sendagi, Bunkyo-ku, Tokyo 113-8603, Japan; 2 Department of Digestive Surgery, Toranomon Hospital, 2-2-2 Toranomon, Minato-ku, Tokyo 105-0001, Japan; 3 Department of Pathology, Toranomon Hospital, 2-2-2 Toranomon, Minato-kuTokyo 105-0001, Japan; 4 Department of Surgery, Nippon Medical School Hospital, 1-1-5 Sendagi, Bunkyo-ku, Tokyo 113-8603, Japan; 5 Department of Pathology, Yokohama City University Graduate School of Medicine, 3-9 Fukuura, Kanazawa-ku, Yokohama 236-0004, Japan

**Keywords:** Duodenal GIST, Skeinoid fiber, Histopathology, Prognosis, Medical examination

## Abstract

**Background:**

Although gastrointestinal stromal tumors (GISTs) are the most common mesenchymal tumors of the gastrointestinal tract, they are very rare. This study evaluated clinical and histopathological characteristics of duodenal GISTs to identify factors useful in predicting prognosis for patients with these tumors.

**Methods:**

A retrospective study was performed on 20 patients who had undergone surgery between 1987 and 2009 for duodenal GISTs. Clinical, histopathological, and immunohistochemical data were evaluated. Survival analyses were conducted using Kaplan-Meier estimates.

**Results:**

In 12 patients (60%), duodenal GISTs were diagnosed incidentally. Eight cases (40%) were classified as high risk grade GISTs. Skeinoid fibers (SkF), which are eosinophilic globular hyaline deposits in the extracellular interstitium of the tumor, were found in 12 patients. Skeinoid fibers were not recognized in 8 cases, and these included 3 cases (37.5%) where tumors recurred after surgery and the patient died. Tumors without SkF were larger (81 ± 92 vs. 23 ± 8 mm, *P* < 0.001) and had a higher mitotic count (224.0 ± 336.6 vs. 0.0 ± 0.0 /50 high-power field, *P* < 0.001) than those with SkF. Survival time was shorter in patients with tumors lacking SkF (52.9 ± 50.7 vs. 108.9 ± 86.5 months, *P* = 0.019).

**Conclusions:**

We have identified clinical and histopathological characteristics that were useful in predicting the prognosis of patients with duodenal GISTs. In this study, 60% of the tumors were found incidentally, SkF were not recognized in tumors from 40% of patients, and all cases of post-operative tumor recurrence and death occurred in this subgroup of patients.

## Background

Gastrointestinal stromal tumors (GISTs) are the most common mesenchymal tumors affecting the gastrointestinal tract [1]. The cells in these tumors show differentiation toward the interstitial cells of Cajal [2]. The majority of GISTs express a growth factor receptor with tyrosine kinase activity termed KIT which is constitutively activated. KIT is the product of the proto-oncogene CD117 (*c*-*kit*) [3]. In GISTs, morphologic features including mitotic count and tumor size have been most acknowledged to be important in predicting malignant tumor behavior [4]. These tumors are common in the stomach (60-70% of cases) and small intestine (30%). However, duodenal GISTs are very rare and constitute less than 5% of all GISTs [5]. There are very few reports describing duodenal GISTs [6-9].

Duodenal and intestinal GISTs contain skeinoid fibers (SkF); eosinophilic globular hyaline deposits found in the extracellular interstitium of the tumor. The ultrastructural appearance of SkF is similar to that of the skein of yarn [10]; however the composition and origin of SkF remains unknown. Several studies [4-7,10,11] suggest that SkF in GISTs are associated with low tumor proliferative activity, but the association between SkF in duodenal GISTs, tumor malignancy and patient prognosis has not been investigated thoroughly.

The aim of this study was to evaluate the clinical and histopathological characteristics of duodenal GISTs, to determine factors useful in predicting patient prognosis.

## Methods

### Clinical background

A retrospective study was performed using clinical and pathological data from 20 patients with duodenal GISTs. Patients who underwent surgery for duodenal GISTs between November 1987 and May 2009 were enrolled in the study; 12 had undergone surgery in Toranomon Hospital and 8 in Nippon Medical School Hospital. The diagnosis of GIST was confirmed after pathological review and positive immunoreactivity for KIT. The diagnosis of GIST was also confirmed in KIT negative tumors based on morphology and positive CD34 immunohistochemical results. Confirmatory mutation studies were not performed. The study population comprised 9 men and 11 women; aged 42 to 79 years (mean 60 ± 12 years). All patients gave written informed consent within an approved ethics process. The study was conducted in accordance with the Declaration of Helsinki.Clinical background, including age, sex, chief complaint, method of GIST detection, location of GIST in the duodenum, and surgical procedures used are detailed in Table 1.

**Table 1:** Characteristics of patients with duodenal GISTs

**Variable**	**Total**	**SkF group**	**non-SkF group**	***P *value**
Number	20	12	8	
Age – yr (mean ± SD)	59.9 ± 11.6	63.6 ± 9.8	54.3 ± 12.3	0.048
Male/Female sex – no. (% male)	9/11 (45)	2/10 (16.7)	7/1 (87.5)	0.003
Presentation reason – no. (%)				
Medical check-up	10 (50%)	6 (50%)	4 (50%)	0.675
Bleeding	3 (15%)	2 (17%)	1 (13%)	0.656
Anemia	5 (25%)	2 (17%)	3 (38%)	0.296
Others*	2 (10%)	2 (17%)	0 (0%)	0.347
Diagnostic method – no. (%)				
Fiber-optic gastroscopy	14 (70%)	7 (58%)	7 (88%)	0.187
Abdominal ultrasonography	2 (10%)	1 (8%)	1 (13%)	0.653
Upper gastrography	2 (10%)	2 (17%)	0 (0%)	0.347
Abdominal computed tomography	1 (5%)	1 (8%)	0 (0%)	0.600
Operation for another gastrointestinal disease	1 (5%)	1 (8%)	0 (0%)	0.600
Therapeutic surgical procedure – no. (%)				
Partial resection	17 (85%)	12 (100%)	5 (63%)	0.049
Distal gastrectomy	2 (10%)	0 (0%)	2 (25%)	0.147
Pancreaticoduodenectomy	1 (5%)	0 (0%)	1 (13%)	0.400
Site of tumor in duodenum – no. (%)				
1st portion of the duodenum	5 (25%)	4 (33%)	1 (13%)	0.307
2nd portion of the duodenum	9 (45%)	5 (42%)	4 (50%)	0.535
3rd portion of the duodenum	4 (20%)	2 (17%)	2 (25%)	0.535
4th portion of the duodenum	2 (10%)	1 (8%)	1 (13%)	0.653
Risk of aggressive tumor behavior (modified AFIP consensus criteria)^12^ – no. (%)				
Very low risk	3	3	0	0.193
Low risk	9	9	0	0.001
High risk	8	0	8	<0.001

Data expressed as mean ± SD, where appropriate. AFIP = Armed Forces Institute of Pathology.

* One patient’s duodenal GIST was detected when she had a gastric smooth muscle tumor resected, and another patient’s duodenal GIST was detected as an incidental finding on a computed tomographic scan of the abdomen performed to screen for metastasis of malignant melanoma.

### Histopathological assessment

The gross features of the duodenal GISTs, including growth pattern and tumor size, were verified from surgical and pathological records. Tumor tissue samples were fixed in 20% neutral buffered formalin, embedded in paraffin, and sectioned at a thickness of 3 μm. Serial sections were stained with hematoxylin and eosin, Elastica van Gieson (EVG) or Elastica-Masson Goldner (EMG), periodic acid and Schiff (PAS) stain, and diastase treated PAS stain. Three authors (T.S., Y.O., and K.O.) assessed each sample for histologic and immunohistochemical evidence of GIST. We examined the specimens 3 times in random order, with the pathologist blinded to the clinical background of the individuals from whom the tumors were removed. Histopathologic evaluations were determined by consensus in the case of inter-pathologist differences. The following histological findings were recorded for each tumor: ulceration of the tumor surface; tumor necrosis; shape of tumor cell (spindled cell pattern, epithelioid cell pattern, and mixed spindle and epithelioid cell pattern); nuclear shape and enlargement; total number of mitoses in 50 high-power fields (HPF; ×40 objective and × 10 ocular lenses), and presence of SkF, on an Olympus BX-50 microscope (Olympus Optical Co., Tokyo, Japan).

### Immunohistochemistry

Indirect immunoperoxidase staining, with or without heat-induced epitope retrieval (autoclaving for 5 min in 10 mmol/L citrate buffer, pH 6.0), was performed using consecutive silane-coated paraffin sections sampled from the tumor-nontumor junction. The target differentiation antigens, visualized using monoclonal and polyclonal antibodies, included smooth muscle markers such as α-smooth muscle actin (SMA) and desmin, and Schwann cell-related markers such as S-100 protein. Immunostaining was used to detect KIT and CD34. To demonstrate proliferative activity, paraffin-resistant Ki-67 antigen was detected immunohostochemically by heating paraffin-embedded tumor tissue and then staining with MIB-1 antibody. The results were evaluated using the following criteria: negative, focally positive (less than 50% of tumor cells showing a positive reaction); diffusely positive (more than 50% of tumor cells stained). The labeling indices of MIB-1 antibody were evaluated by counting positive nuclei in 1000 tumor cells.

### Classification of aggressive behavior in duodenal GISTs

The malignant potential of the tumors was estimated based on tumor size and mitotic count, according to the modified Armed Forces Institute of Pathology (AFIP) consensus criteria for risk stratification of duodenal GISTs [12].

### Statistical analysis

The discontinuous variables, such as observational histopathological results and patient numbers for some clinical parameters, were compared by Fisher’s exact method. The continuous variables, such as clinical data, were evaluated by Wilcoxon rank-sum test. Disease free survival and overall survival were calculated using the Kaplan-Meier method. Univariate analysis was performed using the Log-rank test. Statistical analyses were performed using the SPSS software package (SPSS Inc., Chicago, IL, USA). All data were expressed as the mean ± SD. *P* values < 0.05 were considered significant.

## Results

### Clinical findings, tumor location, and surgery

The baseline clinical characteristics of patients with duodenal GISTs are summarized in Table 1. Gastrointestinal hemorrhage, manifested by hematemesis or melena, was the most frequent symptom. Ten (50%) of patients were asymptomatic and incidentally detected during physical examination for investigation of another disease. In 2 patients, the tumor was incidentally detected during a medical procedure or abdominal surgery performed for another reason. In the first patient the duodenal GIST was detected during resection of a gastric smooth muscle tumor, and in the second patient the duodenal tumor was identified in a computed tomographic scan of the abdomen performed to screen for metastatic malignant melanoma.

Of the 20 duodenal GISTs, 5 were in the 1st portion of the duodenum, 9 in 2nd portion, 4 in 3rd portion, and 2 in 4th portion. Fourteen patients were treated with segmental duodenal resection, 3 cases had tumor resection, 2 cases had distal gastrectomy, and 1 patient had pancreatoduodenectomy. Two patients who had distal gastrectomy performed, had GISTs located in the 1st portion of the duodenum. One of these two cases had a complicated smooth muscle tumor in the pyloric part of stomach. There were no cases of acute abdomen prompting emergency surgery. There were also no neurofibromatosis type 1 patients with small intestinal GISTs. A patient, with a 32 mm diameter duodenal GIST, received 400 mg per day of Imatinib (STI571, Gleevec®) preoperatively for 2 months, but as there was no apparent effect on the tumor, tumor resection was performed.

### Histopathological and immunohistochemical findings

Mean tumor size was 32 ± 27 mm. Ulceration was recognized in 15 cases, and coagulation necrosis of the tumor was seen in 6 cases. Twelve cases were classified as having a spindle cell pattern, 7 cases had an epithelioid cell pattern, and one case had a mixed spindle and epithelioid cell pattern. Nuclear pleomorphism was usually moderate. The mitotic count in the 20 duodenal GISTs ranged from less than 1 to 934 mitotic figures /50 HPFs (mean: 89.6 ± 233 /50 HPFs). The mitotic count was <5 /50 HPFs in 14 cases (70%), and 4 tumors (20%) had more than 50 mitosis /50 HPFs. All 20 cases had documented KIT positivity. Two cases showed KIT positivity in less than 30% of the tumor cells. CD34 reactivity was seen in 14 cases (70%), S-100 was positive in 14 cases (70%), α-SMA was reactive in 14 cases (70%), and 11 cases (65%) were positive for desmin. These immunohistochemical findings were not found to be significantly associated with malignant behavior in duodenal GISTs or patient prognosis. All GISTs had positive reactivity for MIB-1. The MIB-1 index of duodenal GISTs was 1.8 ± 1.2 (range 0.15-60).

### Association of clinical factors and presence of skeinoid fibers

**Figure 1:**
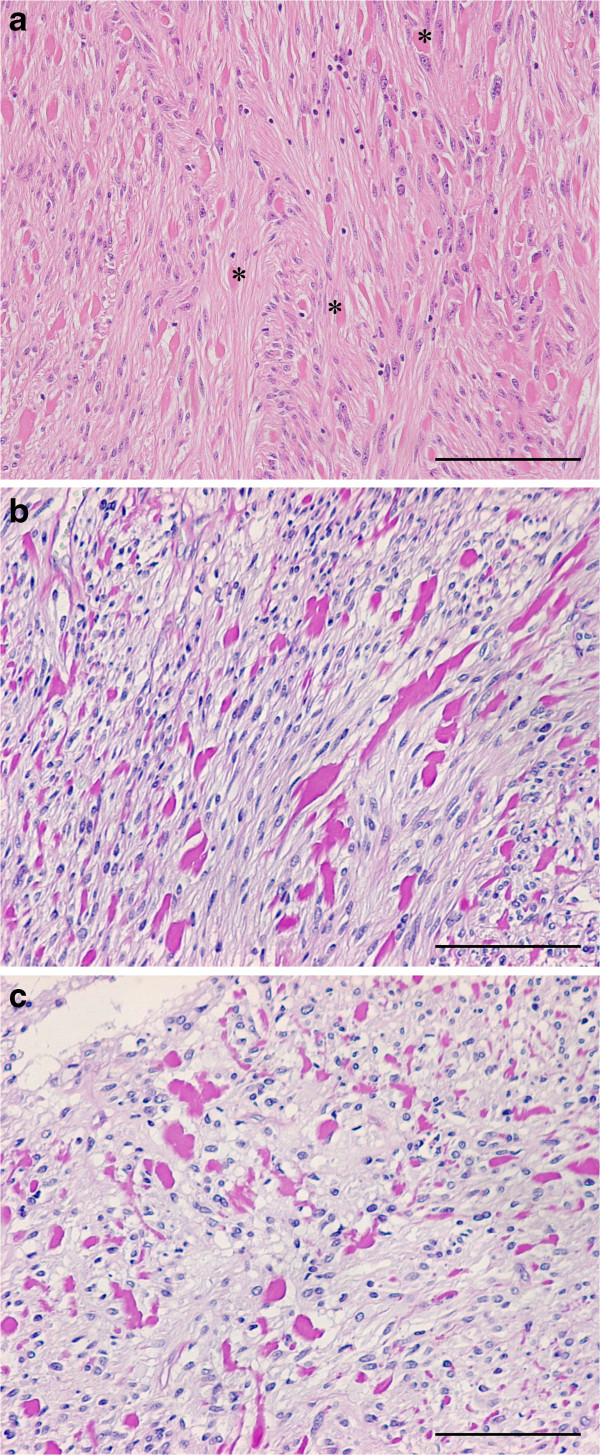
**Histopathological findings in duodenal gastrointestinal stromal tumors. (a)** 61 year old woman without recurrence post-operatively. The tumor, size 33 mm, consists of spindle cells with a fascicular arrangement. Skeinoid fibers (SkF, *) are seen between the spindle cells (hematoxylin and eosin stain). **(b)** and **(c)** 61 year old woman without tumor recurrence post-operatively. The tumor size was 25 mm. Periodic Acid-Schiff reactivity is positive in SkF **(b)** and negative in the diastase digestion test **(c)**. Scale bar = 100 μm in **a**, **b**, and **c**.

Skeinoid fibers, which are variably sized eosinophilic globules, were located between tumor cells and were surrounded by an artifact-like empty halo (Figure 1). These fibers stained positively with PAS stain, and negatively with EVG and EMG stains. Their diastase treated test was also negative. Skeinoid fibers also showed no immunoreactivity with KIT, CD34, α-SMA, desmin, and S-100. These fibers were recognized in 12 duodenal GISTs (SkF group), with the other 8 tumors lacking these fibers (non-SkF group). Clinical differences between these 2 groups are shown in Table 1 and pathological differences in Table 2. The GISTs size in the non-SkF group was significantly larger than in the SkF group (81 ± 92 vs. 23 ± 8 mm, *P* < 0.001). All tumors in the non-SkF group were ulcerated. Tumor necrosis was present in 6 tumors: all in the non-SkF group. Cellular shape (spindle or epithelioid cell pattern) had no correlation with presence of SkF. The mitotic count and MIB-1 index in the non-SkF group was also greater than that in SkF group (224.0 ± 336.6 vs. 0.0 ± 0.0 /50 HPF, *P* < 0.001; and 26.3 ± 20.3 vs. 1.8 ± 1.2, *P* < 0.001, respectively).

**Table 2:** **Pathological and immunohistochemical findings in duodenal GISTs**, **and a list of primary antibodies used in diagnosis**

**Variable**	**Dilution, Source of Antibodies**	**Total**	**SkF group**	**non-SkF group**	***P *value**
Number		20	12	8	
Tumor size – mm		46.5 ± 63.4	23.3 ± 8.2	81.2 ± 92.2	<0.001
Spindle type cell - no. (%)		13 (65%)	8 (67%)	5 (63%)	0.608
Necrosis - no. (%)		6 (30%)	0 (0%)	6 (75%)	<0.001
Ulceration - no. (%)		15 (75%)	7 (58%)	8 (100%)	0.051
Mitosis - no. per 50 HPF		89.6 ± 233.3	0.0 ± 0.0	224.0 ± 336.6	<0.001
Mib-1 index -%	1:200, autoclave; MBL	11.6 ± 17.4	1.8 ± 1.2	26.3 ± 20.3	<0.001
KIT - no. (%)	1:100, autoclave; Dako	20 (100%)	12 (100%)	8 (100%)	0.999
CD34 - no. (%)	1:100, autoclave; Ventana	14 (70%)	9 (75%)	5 (63%)	0.455
S-100 - no. (%)	1:1000; Dako	14 (70%)	9 (75%)	5 (63%)	0.455
α-smooth-muscle actin - no. (%)	1:200, autoclave; Dako	14 (70%)	9 (75%)	5 (63%)	0.455
Desmin - no. (%)	1:40, autoclave; Dako	11 (55%)	8 (67%)	3 (38%)	0.205

Data expressed as mean ± SD, where appropriate.

### Classification of aggressive behavior in duodenal GISTs

**Figure 2:**
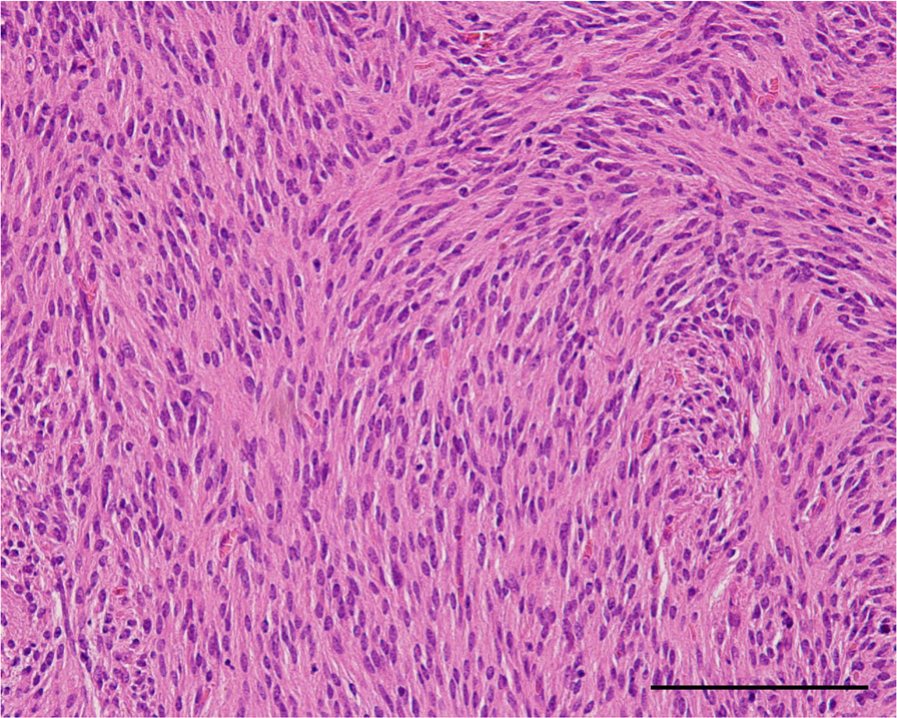
**Histopathological features of a duodenal gastrointestinal stromal tumor.** 44 year old man with tumor recurrence after resection; recurrence detected as hepatic metastasis and peritoneal disseminations. Tumor size was 110 mm. Tumor showing proliferation of spindle cells arranged in interlacing bundles. There were no skeinoid fibers in this tumor (hematoxylin and eosin stain). Scale bar = 00 μm.

According to the modified AFIP consensus criteria for risk stratification of GISTs [12], in this study there were 3 tumors in the very low risk category, 9 in the low risk, and 8 in the high risk. All patients with tumors in the SkF group were classified with either a very low or a low risk of aggressive tumor behavior, and those in the non-SkF group with high risk of aggressive tumor behavior Figure 2.

### Survival and tumor recurrence

**Figure 3:**
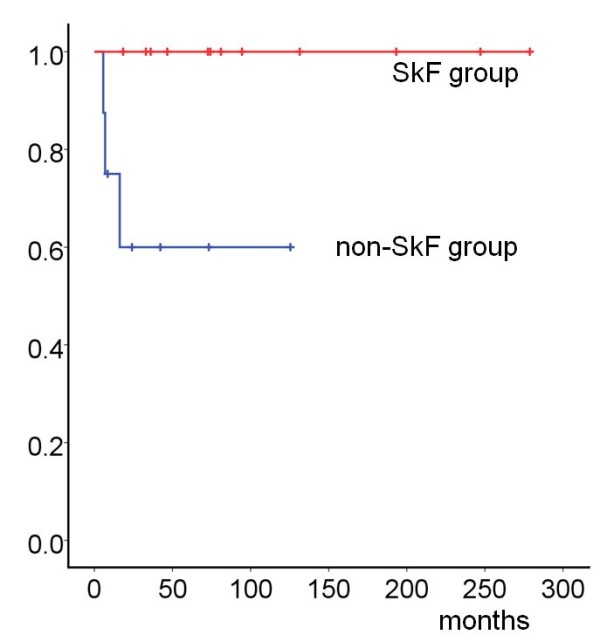
**Disease**-**specific survival after resection of duodenal GIST.** Patients without skeinoid fiber (SkF, n = 8) had significantly worse survival than those with SkF (n = 12, *P* = 0.019).

Seventeen (85%) patients were alive with no evidence of disease for a median survival time of 6.0 years. Overall tumor-specific mortality was 15% (3 of 20) in patients with follow-up. Of these patients, one was a woman and two were men. Their duodenal GISTs recurred a mean of 9.6 ± 5.8 months after the initial operation. One patient had liver metastasis and two patients had abdominal metastasis. They died due to this disease a mean of 49.9 ± 68.3 months following recurrence, all being in the no SkF group. We performed a combination analysis using the Kaplan-Meier method (Figure 3) and Log-rank test, both of which showed that the non-SkF group had a significantly shorter survival time (52.9 ± 50.7 vs. 108.9 ± 86.5 months, *P* = 0.019).

## Discussion

The present study showed that duodenal GISTs, without any metastasis or local invasion, may be completely cured by early resection. In this study, 12 cases had SkF in the extracellular interstitium between tumor cells. In our observation, all tumors containing SkF were of low grade malignancy; the prognosis for patients with duodenal GISTs with SkF was significantly better than for those with tumors lacking SkF.

Predicting the malignant potential of GISTs may be difficult, and there is currently no consensus on risk estimates for metastasis to the liver or peritoneum, or for patient mortality. For example, small tumors with a low mitotic rate may still metastasize. Further, GISTs in the small intestine seem to behave more aggressively than gastric GISTs of similar size and mitotic activity [12,13]. Miettinen et al. [7] proposed that duodenal GISTs whose mitotic count exceeded 2 mitoses /50 HPFs be regarded as high grade malignancies. However, prominent nuclear pleomorphism and hyperchromasia are rare in duodenal GISTs. Mortality also occurs in patients whose duodenal GISTs are large, even if the mitotic count is low, so that tumor size is the most important factor in grading malignancy of duodenal GISTs. Early diagnosis and resection of duodenal GISTs, improves patient prognosis.

Japanese proactively seek medical examination including fiber-optic gastroscopy (FGS) and abdominal ultrasonography. This enables early detection of duodenal GISTs. In our study, 12 of 20 duodenal GISTs (60%) were found incidentally, and 5 of these 12 cases were detected endoscopically. In addition, 7 cases (35%) were recognized by FGS performed to investigate the cause of gastrointestinal bleeding or the cause of anemia. Therefore, 12 patients (60%) had their duodenal GISTs detected by endoscopy. In our study, 60% of patients were at very low or low risk from aggressive tumor behavior according to modified AFIP consensus criteria for risk stratification of GISTs, in which the main contributor to risk is tumor size [12]. Gastrointestinal stromal tumors in Japanese patients have been reported to contain similar genetic mutations to GISTs in patients from other countries [14]. The early detection of GISTs allows prevention of metastasis or local tumor invasion and improved prognosis for patients with duodenal GISTs.

The composition of SkF is still unknown. These fibers were present between tumor cells and were surrounded by an artifact-like empty halo; suggesting SkF were composed of solider elements than tumor cells. Skeinoid fibers appear as tangles of fibers similar to regular collagen, with cross-banding of 41–45 nm periodicity, and their length are shorter than normal collagen fibers [15]. Some studies reported that SkF contain type VI collagen [10,15,16]. We regard SkF to consist of a type of polysaccharide because they stain positively with PAS and negatively with the diastase digestion test (Figure 1b and c). They also show no immunoreactivity with KIT, CD34, α-SMA, desmin, or S-100 immunostaining.

In this study, all duodenal GISTs with SkF in the extracellular interstitium were categorized as very low or low risk according to the modified AFIP criteria for risk stratification of GISTs, and 8 cases lacking SkF were categorized as high risk due to their size and number of mitoses. These results suggest SkF are associated with less aggressive behavior in duodenal GISTs. We hypothesize that the material SkF are composed of has been secreted from tumor cells and retained in the extracellular space. Highly malignant GISTs grow rapidly, suggesting that secretion of the SkF material does not have time to accumulate in rapidly proliferating highly malignant duodenal GISTs.

Genetic research has shown that GISTs with spindle type cells and those with SkF contained a *c*-*kit* mutation, and GISTs with epithelioid type cells and those without SkF had an internal tandem duplication in exon 11 of *PDGFRA* mutation [17]. This led to the hypothesis that SkF may be a product of *c*-*kit*. However in our study, there was no relationship between tumor cell type and presence or absence of SkF, and SkF showed negative reactivity on KIT immunostaining. *PDGFRA* encodes a cell surface tyrosine kinase receptor for members of the platelet-derived growth factor family. It is located near to, and is structurally similar to, *c*-*kit*, leading to speculation of a similar origin for both genes. Therefore, this study suggests there may be no association between either gene and SkF.

In this study, patients with duodenal GISTs containing SkF experienced better overall survival than patients with duodenal GISTs lacking SkF (100% vs. 62.5%). All 3 patients that died in the follow up period in this study suffered tumor recurrence within half a year, and their original tumor sizes were over 50 mm. No tumor recurrence or metastasis occurred in patients in this study, when tumors were small and were resected completely. Although the current classification of GISTs is appropriately based on tumor size and number of cellular divisions counted, adding the speed of tumor growth as an indicator of tumor malignancy may further improve the current risk classification system. Miettinen et al. [7] detected SkF in 78 (53%) of 148 patients with duodenal GIST, and 70 of these cases were classified as very low or low malignant potential. This is similar to our results, however no statistical evaluation of the correlation between SkF and survival was performed. To our knowledge, the present study is the first to evaluate the association of SkF with prognosis in duodenal GISTs.

The major limitation of this present study was the small number of patients with duodenal GISTs, although our study does describe 20 cases with adequate follow up seen in 2 institutes. The largest study on 156 cases with duodenal GISTs was performed at the Armed Forces Institute of Pathology (and the Haartman Institute of the University of Helsinki) between 1970 and 1996 [7]. The second-largest study had 22 patients [6], and the third-largest one had only 20 patients [8]. Thus, as a general trend, cases with duodenal GISTs are few in number because the duodenum is a short organ. Duodenal GISTs in Japanese are able to be detected early because of the social background of proactive medical examination, and this early diagnosis may facilitate research into the early biological characteristics of this relatively unusual tumor. A multicenter study on duodenal GISTs must thus shed further light on the biologic behavior of this tumor.

## Conclusions

We have identified clinical and histopathological factors that are useful in predicting prognosis in patients with duodenal GISTs. In this study, 60% of the tumors were found incidentally. Skeinoid fibers were not recognized in tumors from 40% of patients; all cases of post-operative tumor recurrence and patient death occurred in patients with duodenal GISTs lacking SkF.

### Consent

Written informed consent was obtained from the patient for publication of this report and any accompanying images.

## Abbreviations

EMG: Elastica-Masson Goldner; EVG: Elastica van Gieson; GIST: Gastrointestinal stromal tumors; HPF: High-power fields; PAS: Periodic acid and Schiff; SkF: Skeinoid fiber; SMA: Smooth muscle actin.

## Competing interests

The authors declare that they have no competing interests.

## Authors’ contributions

ST, UM, NY, and WG designed and conducted the study. ST, OY, and OK analyzed the data, especially pathological evaluation. HM, UH, and MK helped to write the manuscript. WG is the principal investigator, revised and edited the manuscript. All authors approved the final manuscript.
